# The Story of the Insane from Year to Year

**Published:** 1892-01-23

**Authors:** 


### The Story of the Insane from Year to Year.

(Fourth Series).

### CHESTER COUNTY ASYLUM AT UPTON.

On January 1st, 1890, there were 599 patients on the books of this Asylum, and this number was increased by 5 at the close of the year. The admissions reached 150; the recoveries 83, and the deaths 58. The two latter yield respectively the percentages of 55·3 and 9·5. Intemperance in drink was the cause of the insanity in 17 cases, and hereditary influence in 23. Brain diseases were fatal in 20 cases, and phthisis in 4. The Visiting Committee say they have "much pleasure to state that great credit is due to Dr. Davidson and the staff generally for the economical and efficient manner in which the Asylum has been managed, and that with one exception, no charge has been brought against any of the attendants for irregularity of conduct." The Commissioners in Lunacy think that the increased duties imposed on the medical staff of Asylums by the recent Act will require another assistant medical officer at this Asylum. There is a balance of £771 in favour of the farm; but the no-rent system is in vogue, and no prices are stated for the articles supplied from the farm to the Asylum. The weekly cost per head is a fraction under 6s. 8½d.

### THE CUMBERLAND ASYLUM.

A slight decrease in the number of patients is shown by the statistics on Table I. The Asylum began the year with the names of 579 patients on the books, and it ended the year with 571. The admissions reached 140; the recoveries 73, and the deaths 79. The percentage of recoveries is high—48·6, and the death-rate is 8·3 on the average number resident. Hereditary taint caused the disease in 37 cases of the 140, and drink was the cause in 18. Of the deaths 15 were from brain diseases, and 8 from phthisis. Dr. Campbell thinks that had more carefully compiled statistics existed for many years as to the potency of hereditary influence, we should by this time have had more accurate knowledge of certain matters; and he proceeds, "I believe that hereditary predisposition from the maternal side is more baneful than from the paternal; that the predisposition derived from melancholic states with suicidal tendencies is a more transmissible insane element than the nerve flaws which result in cases of mere excitement, and that the unknown condition which produces epileptic insanity is, of all others, one of the most dangerous legacies." It is certain that the more careful the statistics are the greater will be their use, at least from a scientific standpoint; but the broad fact of the strongly hereditary nature of insanity has been known long enough, and the effect of the knowledge may be estimated from the figures we quoted at the beginning of this notice. Terrible as are these statistics of heredity in insanity, they are most likely very far short of the truth, and if that truth were known Dr. Campbell would have to return not 37 of his last years' admissions as hereditary, but the number would be nearer 74, if it did not exceed it. The Visiting Committee say they are "perfectly satisfied with the management and superintendence of the Asylum, and the efforts which are made by Dr. Campbell and his assistants to effect the recovery of the patients." The average weekly cost per head per week was 9s. 4d.

### THE HEREFORD COUNTY ASYLUM.

The numbers were almost stationary during the year 1890. There were 373 patients in the Asylum on the 1st of January, and 376 on the last day of December. Sixty-nine patients were admitted; 27 recovered, and 23 died. The recoveries, calculated in the usual way, were 39·1 per cent, and the deaths were 6 2. The recoveries are about the average in County Asylums, and the deaths are considerably lower. Of the admissions, 7 were driven insane by drink, and 8 owing to hereditary influence. Brain disease accounts for 11 of the deaths, and phthisis for only 1. Dr. Chapman tells us the admissions were more than usually unfavourable as regards prospect of recovery, and we can readily believe this when we hear that "10 were idiots from birth; 21 had been insane more than twelve months, and 8 were over 70 years of age. More than half the admissions, therefore, presented very faint possibilities of recovery. Indeed only 19 suffered from a first attack of less than three months' duration, and of these several suffered from hopeless brain disease." Perhaps, however, if Dr. Chapman had the unspeakable boon of a hospital for acute cases at Hereford, he would cure 60 per cent. of the above. At least, this is what we have been seriously told would result from the hospital treatment. What does Dr. Chapman say about it? The Visiting Committee "desire to express their satisfaction with the continued successful management of the Asylum and all belonging to it by the Medical Superintendent." The farm accounts show a balance of £499. Weekly cost per head is 8s. 11⅝d.

### NORTHUMBERLAND COUNTY ASYLUM.

A decrease from 543 patients to 532 is to be noted during the year 1890. The admissions reached 114; the recoveries 56, and the deaths 56. The percentage of recoveries stands at 51·6, and the deaths at 10·3. Drink was the cause of the insanity in 21 cases, and hereditary influence in 33. Brain diseases killed 20, and consumption 9. An out-patients' department has been started at this Asylum, and we are not surprised to find that the result is rather disappointing. Indeed one does not very clearly see from what direction good is likely to come from these out-patients' departments. Dr. McDowall has the following sentence or two on the New Lunacy Bill: "The Lunacy Act has provoked such unanimous condemnation, and is so vexatious in its provisions that it is much to be regretted that it has secured a place among the statutes. It greatly increases the duties of the Medical Superintendent and of the Clerk, without benefiting the patients in the slightest degree. In some Asylums it has already been necessary to increase the number of officers." Dr. McDowall is doubtless aware of what we stated in a previous notice, that the best Acts of Parliament have been those which repealed other Acts. Here then is a fine opportunity for a really good Act. Lectures are given on ambulance work and nursing in this Asylum. The Visiting Justices say, "The management of the Asylum, under the efficient Superintendence of Dr. McDowall, and the conduct of the officers and servants, continue to give complete satisfaction to your Committee." The balance in favour of the farm is a little over £914. As usual we see nothing charged for rent nor interest on capital, nor patients' labour. The weekly cost of maintenance is 9s. 5⅜d. per head.

